# Non-Coated Rituximab Induces Highly Cytotoxic Natural Killer Cells From Peripheral Blood Mononuclear Cells *via* Autologous B Cells

**DOI:** 10.3389/fimmu.2021.658562

**Published:** 2021-05-25

**Authors:** Chao Niu, Yongchong Chen, Min Li, Shan Zhu, Lei Zhou, Dongsheng Xu, Zhaozhi Li, Jianting Xu, Wei Li, Yufeng Wang, Jiuwei Cui

**Affiliations:** ^1^ Department of Cancer Center, The First Hospital of Jilin University, Changchun, China; ^2^ Department of Translational Medicine, The First Hospital of Jilin University, Changchun, China; ^3^ Cancer Institute, The First Hospital of Jilin University, Changchun, China

**Keywords:** rituximab, NK cell, B cell, cytotoxicity, tumor therapy, antibody-dependent cell-mediated cytotoxicity

## Abstract

Natural killer (NK) cells are becoming valuable tools for cancer therapy because of their cytotoxicity against tumor cells without prior sensitization and their involvement in graft-versus-host disease; however, it is difficult to obtain highly cytotoxic NK cells without adding extra feeder cells. In this study, we developed a new method for obtaining highly cytotoxic NK cells from peripheral blood mononuclear cells (PBMCs) independently of extra feeder cell addition using rituximab not coated on a flask (non-coated rituximab). We found that rituximab could promote both the activation and expansion of NK cells from PBMCs, irrespective of being coated on a flask or not. However, NK cells activated by non-coated rituximab had much greater antitumor activity against cancer cells, and these effects were dependent on autologous living B cells. The antibody-dependent cellular cytotoxicity effect of NK cells activated by non-coated rituximab was also more substantial. Furthermore, these cells expressed higher levels of CD107a, perforin, granzyme B, and IFN-γ. However, there was no difference in the percentage, apoptosis, and cell-cycle progression of NK cells induced by coated and non-coated rituximab. Non-coated rituximab activated NK cells by increasing AKT phosphorylation, further enhancing the abundance of XBP1s. In conclusion, we developed a new method for amplifying NK cells with higher antitumor functions with non-coated rituximab *via* autologous B cells from PBMCs, and this method more efficiently stimulated NK cell activation than by using coated rituximab.

## Introduction

Natural killer (NK) cells are important innate immune cells that play essential roles in tumor surveillance (1, 2). The function and number of NK cells in patients with cancer are usually diminished compared to healthy individuals (3, 4). The abundance of NK cells in tumors is positively correlated with prognosis (5–7). Adoptive NK cell therapy, especially donor NK cell infusion after allogeneic stem cell transplantation, has exhibited impressive clinical responses (8–11). The clinical outcomes of NK cell therapies are closely correlated with their antitumor cytotoxicity (12, 13). Therefore, enhancing the antitumor function is essential for improving the clinical efficacy of NK cell therapy.

Feeder cells and cytokines are frequently used to enhance NK cell activity (14). Feeder cells can help NK cells obtain powerful antitumor functions and can stimulate their proliferation (15). Genetically modified K562 cells, a lymphoblastoid cell line infected with Epstein–Barr virus, are among the cancer cell lines commonly used as feeder cells (16–19). Feeder cells (which are typically cancer cells) increase the clinical risks of adoptive NK cell therapies. Cytokines, such as interleukin (IL)-2, IL-15, IL-7, IL-12, and IL-18, can also cause NK cells to acquire powerful antitumor functions and undergo rapid proliferation (20–24). Cytokines are frequently used to amplify NK cells; however, their effects are weaker than feeder cells. Therefore, other methods for activating NK cells need to be developed.

Rituximab, a chimeric human-mouse monoclonal antibody (mAb) that targets CD20, can activate NK cells and cause B-cell death by antibody-dependent cell-mediated cytotoxicity (ADCC) (25, 26). In this study, we investigated the use of B cells as feeder cells to activate and expand NK cells through rituximab-mediated ADCC. We also explored whether the methodology in which rituximab was applied (coated or non-coated on flasks) could significantly affect the antitumor functions of NK cells.

## Materials and Methods

### Antibody Coating on Flasks

Clinical rituximab (Roche, Basel, Switzerland) was diluted with double-distilled water at concentrations of 0 μg/mL, 0.25 μg/mL, 0.50 μg/mL, 0.75 μg/mL, 1 μg/mL, 2 μg/mL, and 4 μg/mL. Anti-CD16 mAb (3G8, cat # PNIM0813; Beckman Coulter, Brea, CA, USA) and human IgG1 Isotype Control (cat # ALX-804-133; Enzo Life Sciences, Farmingdale, NY, USA) were diluted to 1 μg/mL. The antibodies were then added to T25 flasks (cat # 353014; BD FALCON, Tewksbury, MA, USA) at 5 mL per flask and incubated at 37°C for 4 h. After washing once with phosphate-buffered saline (PBS), the flasks were ready for culturing NK cells.

### NK Cell Culture

Peripheral blood mononuclear cells (PBMCs) were isolated from the blood of healthy donors by Ficoll (Axis-Shield PoC AS, Oslo, Norway) gradient density centrifugation. This procedure was carried out with the approval of the ethics committee of the First Hospital of Jilin University. B cells were isolated using CD20 Microbeads (Miltenyi Biotec, Bergisch Gladbach, Germany). PBMCs (with or without B cells) were adjusted to a concentration of 2 × 10^6^ cells/mL using Aly505 medium (Cell Science & Technology Institute Inc., Yamagata, Japan) containing 5% autologous serum, 600 IU/mL IL-2, and 10 ng/mL IL-15 (both from Miltenyi Biotec). The cells were then added to antibody-coated or non-coated flasks to induced and expanded NK cells. For the NK cells cultured in non-coated rituximab flasks, different concentrations of rituximab (0 μg/mL, 0.25 μg/mL, 0.50 μg/mL, 0.75 μg/mL, 1 μg/mL, 2 μg/mL, and 4 μg/mL) were added directly to the medium at the beginning of culture. In all rituximab treatment groups, anti-CD161 antibodies (HP-3G10, cat # 339902; BioLegend, San Diego, CA, USA) were added to the medium at a final concentration of 1 μg/mL on the first day of culture. After incubation for 3 d, media was replaced with fresh media containing 600 IU/mL IL-2 and 10 ng/mL IL-15. Every 2-3 d, half of the medium was replaced with fresh medium containing 600 IU/mL IL-2 and 10 ng/mL IL-15. NK cells were also expanded with irradiated K562-IL-21 as feeder cells, as previously described (19). After culturing for 14 d, NK cell function was evaluated.

To determine whether living B cells are necessary for expanding and activating NK cells with non-coated rituximab, B cells and NK cells were sorted from PBMCs by CD20 Microbeads and MACSxpress^®^ NK Cell Isolation Kit (Miltenyi Biotec). Living and fixed B cells (treated with 4% formaldehyde) were then incubated with NK cells according to the ratio of B cells and NK cells in PBMCs with either rituximab or nivolumab (OPDIVO, Bristol Myers Squibb, New York, NY, USA) for 3 d. Then the cells were transferred to the culture medium without antibodies and culture continued. After 14 d, the cells were harvested to evaluate NK cell cytotoxicity.

### Analysis by Flow Cytometry

To determine the purity and the surface markers of naïve and induced NK cells, the cells were adjusted to 1 × 10^6^ cells/mL and stained with CD3-PerCP (SK7, cat # 347344), CD56-PE (B159, cat # 555516) or CD56-FITC (B159, cat # 562794), CD57-FITC (HNK-1, cat # 340706), NKG2D-APC (1D11, cat # 558071), KIR3DL1-APC (DX9, cat # 564103), KIR2DL2/3-PE (CH-L, cat # 559785), and KIR2DL1-PE (HP-3E4, cat # 556063) (all from BD Biosciences, San Jose, CA, USA) and NKG2A-PE (S19004C, cat # 375104; BioLegend) for 15 min at 23°C in the dark. The cells were washed twice with PBS and detected using a BD FACS Aria II flow cytometer (BD Biosciences) or ACEA NovoCyte D3000 flow cytometer (ACEA Biosciences Inc., San Diego, CA, USA). The data were analyzed using FlowJo software (Tree Star, Ashland, OR, USA).

CD16-FITC (NKP15, cat # 335035), interferon (IFN)-γ-PE (4S.B3, cat # 554552), granzyme B-PE (GB11, cat # 561142), perforin-PE (δG9, cat # 556437), and CD107a (H4A3, cat # 641581) assays (all from BD Biosciences) were carried out as previously described (27, 28). Apoptosis was measured using the Annexin V-PE/7-AAD Apoptosis Detection Kit (eBioscience, San Diego, CA, USA), as per the manufacturer’s instructions. To analyze cell-cycle progression, induced NK cells were collected and labeled with propidium iodide.

### NK Cell Cytotoxicity

The cytotoxicity of NK cells was determined by performing the calcein-release test (Dojindo Laboratories, Kumamoto, Japan) as previously reported (28). Target cancer cells were collected and adjusted to a concentration of 1 × 10^6^ cells/mL with PBS and then incubated with 1 μM calcein-AM at 37°C. After 30 min, the target cells were washed twice with PBS and adjusted to a concentration of 5 × 10^4^ cells/mL in RPMI-1640 medium (Gibco, ThermoFisher Biochemical Products (Beijing) Co., Ltd, Beijing, China) containing 5% fetal bovine serum. The effector cells were adjusted to a concentration of 2.5 × 10^5^ cells/mL using the same medium. To detect direct killing by NK cells, 100 μL target cells and 100 μL effector cells were added into the same wells of a 96-well plate. For ADCC experiments, except for the target cells and effector cells mentioned above, rituximab was added to the medium at a final concentration of 10 μg/mL. To determine the minimum release, 100 μL target cells and 100 μL medium were added to the 96-well plates. To determine the maximum release, 100 μL target cells and 100 μL medium containing 0.4% TritonX 100 were added into one well of the 96-well plate.

After a 4 h incubation at 37°C, 100 μL supernatants were transferred to dark 96-well plates and cytotoxicity was measured with a BioTek Synergy HT Microplate Reader (BioTek Instruments, Winooski, VT, USA). The specific cytotoxicity was calculated as follows: specific lysis (%) = [(experimental release – minimum release)/(maximum release – minimum release)] × 100%.

### Western Blot Assays

Western blotting was performed using mouse mAbs against AKT, p-AKT, or XBP1s (all from Cell Signaling Technology, Danvers, MA, USA), appropriate secondary antibodies, and an Enhanced Chemiluminescence substrate reagent kit (Beijing Labgic Technology Co., Ltd., Beijing, China).

### Statistical Analyses

Data were analyzed by one-way or two-way analysis of variation (ANOVA) or two-tailed paired t-test using GraphPad Prism 8 software (GraphPad Software, San Diego, CA, USA).

## Results

### NK Cells Induced by Non-Coated Rituximab Have Much Stronger Antitumor Function

**Figure 1:**
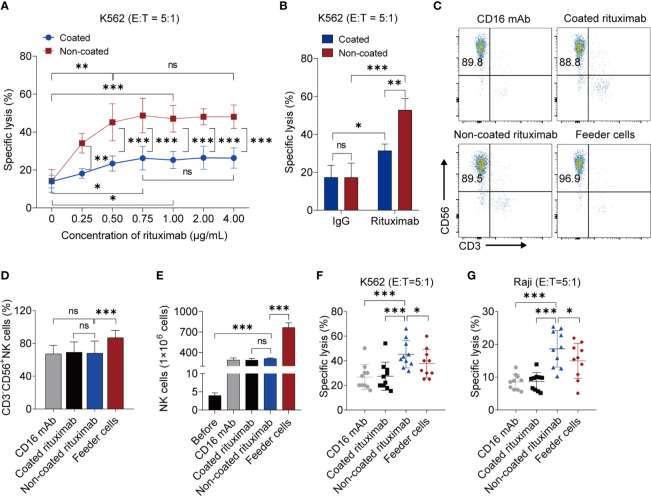
NK cells induced by non-coated rituximab have stronger antitumor activity. **(A)** Titration of rituximab for NK cell expansion. NK cells were expanded with different concentration of coated and non-coated rituximab for 3 d and then cultured in media without rituximab for 11 d. The cytotoxicity of induced NK cells was determined by calcein-release assay. n = 5 donors, ns: not significant, *p* > 0.05, *p < 0.05, **p < 0.01, ***p < 0.001; two-way ANOVA and two-tailed paired t-test. **(B)** Cytotoxicity of NK cells induced by rituximab and human IgG1 Isotype Control. n = 5 donors, ns, not significant, *p* > 0.05, *p < 0.05, **p < 0.01, ***p < 0.001; two-way ANOVA. **(C)** Representative flow cytometry analysis of the percentage of CD3^−^CD56^+^ NK cells induced by coated CD16 mAb, coated rituximab, non-coated rituximab, and K562-IL21 feeder cells. **(D)** Graph showing the percentages of NK cells induced by four different methods for 14 d. n = 10 donors, ns: not significant, *p* > 0.05, ***p < 0.001; one-way ANOVA. **(E)** Comparative analysis of NK cell number before and after induction by four different methods. n = 5 donors, ns: not significant, *p* > 0.05, ***p < 0.001; one-way ANOVA. **(F, G)** Comparative analysis of NK cell cytotoxicity before and after induction by four methods. NK cell cytotoxicity was determined by calcein-release assay. n = 10 donors, *p < 0.05, ***p < 0.001; one-way ANOVA.

We used different concentrations of rituximab to activate NK cells. When the rituximab concentration was below 0.75 μg/mL, the cytotoxicity of NK cells induced by coated rituximab increased with increasing rituximab concentration. However, there was no statistical difference in the cytotoxicity of NK cells among the 0.75 μg/mL, 1 μg/mL, 2 μg/mL, and 4 μg/mL treated groups. This indicates that concentrations of coated rituximab greater than or equal to 0.75 μg/mL do not affect the antitumor function of NK cells. With non-coated rituximab, the cytotoxicity of NK cells cultured with 0.50 μg/mL non-coated rituximab was better than those of 0 μg/mL and 0.25 μg/mL coated rituximab. However, there was no difference with the 0.50 μg/mL, 0.75 μg/mL, 1 μg/mL, 2 μg/mL, and 4 μg/mL non-coated rituximab groups. These results indicated that when the rituximab concentration was greater than or equal to 0.50 μg/mL, the antitumor function of NK cells did not change with the increase of rituximab concentration. (**Figure 1A**). Therefore, in the following experiments, 1 μg/mL rituximab was used to remove differences caused by different concentrations of rituximab. Under the same concentration of rituximab, the antitumor activity of NK cells obtained in the non-coated rituximab group was always significantly stronger than the coated rituximab group (**Figure 1A**). We also found that the cytotoxicity of NK cells induced by rituximab was stronger than NK cells induced by control IgG-isotype antibody, regardless of whether the antibodies were coated or not (coated: 31.44% ± 3.50% *vs.* 17.35% ± 6.37%, *p* < 0.05; non-coated: 52.85% ± 6.06% *vs.* 17.35% ± 7.49%, *p* < 0.001; **Figure 1B**).

To compare the functions between NK cells induced by non-coated rituximab and NK cells induced by previously established methods, a comparative experiment was carried out. Although, the purity and the number of NK cells induced by non-coated rituximab were lower than NK cells induced by feeder cells, there was no difference in the purity and numbers of NK cells, respectively, obtained by non-coated rituximab, coated rituximab, and coated CD16 mAb. (**Figures 1C–E**). More importantly, the cytotoxicity of NK cells obtained by our method was significantly stronger than NK cells cultured with feeder cells and coated CD16 mAb, regardless of the presence of NK cell-sensitive K562 cells (45.31% ± 11.11% *vs.* 37.72% ± 11.46%, *p* < 0.05 and 45.31% ± 11.11% *vs.* 28.86% ± 10.27%, *p* < 0.001, respectively; **Figure 1F**) or NK cell-resistant Raji cells (18.61% ± 5.56% *vs.* 14.96% ± 5.37%, *p* < 0.05 and 18.61% ± 5.56% *vs.* 8.51% ± 2.36%, *p* < 0.001, respectively; **Figure 1G**).

### Non-Coated Rituximab Induced Highly Cytotoxic NK Cells From PBMCs *via* Autologous Living B Cells

**Figure 2:**
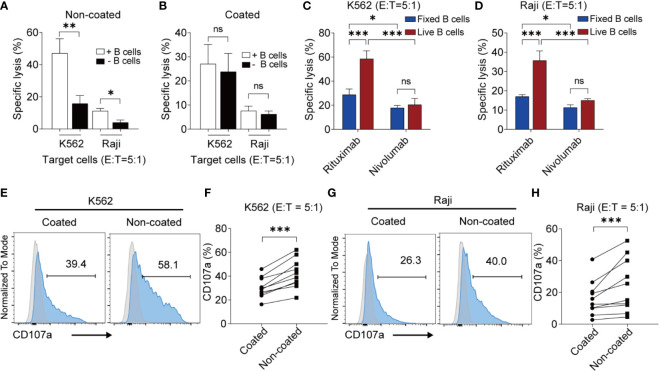
Non-coated rituximab induced highly cytotoxic NK cells from peripheral blood mononuclear cells *via* autologous living B cells. **(A, B)** Cytotoxicity of NK cells induced from PBMCs with and without B cells. PBMCs (with and without B cells) were used to expand NK cells with coated or non-coated rituximab. After 14 d in culture, cells were harvested and cytotoxicity was determined by calcein-release assay. n = 5 donors, ns, not significant, *p* > 0.05, *p < 0.05, **p < 0.01; two-tailed paired t-test. **(C, D)** Cytotoxicity of NK cells induced by living and fixed (dead) B cells. B cells and NK cells were sorted from PBMCs, then living and fixed B cells were incubated with NK cells according to the ratio of B cells and NK cells in PBMCs with rituximab or nivolumab. After 14 d in culture, cells were harvested and cytotoxicity was determined by calcein-release assay. n = 3 donors, ns: not significant, ***p < 0.001; two-way ANOVA. **(E, G)** Representative flow cytometry analysis of the percentage of CD107a^+^ NK cells incubated with two different cancer cell lines. NK cells cultured with coated or non-coated rituximab were incubated with K562 or Raji cells for 4 h, followed by quantification of CD107a^+^ cells. **(F, H)** Statistical analyses of CD107a^+^ NK cells. NK cells induced with coated or non-coated rituximab were incubated with K562 or Raji cells for 4 h, followed by quantification of CD107a^+^ cells. n = 10 donors, ***p < 0.001; two-tailed paired t-test.

To further determine whether this effect was associated with B cells, we depleted the B cells from the NK cell culture system using a B cell isolation kit. The cytotoxicity of NK cells induced by non-coated rituximab decreased sharply after the removal of B cells, regardless of the presence of K562 (47.13% ± 9.03% *vs.* 15.79% ± 5.01%, *p* < 0.01) or Raji cells (11.16% ± 1.60% *vs.* 4.04% ± 1.55%, *p* < 0.05) (**Figure 2A**). However, the cytotoxicity of NK cells induced by coated rituximab was not affected by the presence or absence of B cells (K562 cells: 27.07% ± 8.05% *vs.* 23.80% ± 7.57%, *p* > 0.05; Raji cells: 7.60% ± 1.98% *vs.* 6.18% ± 1.33%, *p* > 0.05; **Figure 2B**). To study whether living B cells are necessary for NK cell activation by non-coated rituximab, we expanded and activated NK cells with living and fixed B cells and either rituximab or nivolumab. After B cells were fixed, although the antitumor effect of NK cells activated by the rituximab group was stronger than that of the nivolumab group (K562 cells: 28.75% ± 4.75% *vs.* 17.80% ± 2.13%, *p* < 0.05, **Figure 2C**; Raji cells: 17.00% ± 1.00% *vs.* 11.30% ± 1.47%, *p* < 0.05, **Figure 2D**), it was still significantly lower than that of rituximab combined with living B cells group (K562 cells: 28.75% ± 4.75% *vs.* 58.47% ± 6.78%, *p* < 0.001, **Figure 2C**; Raji cells: 17.00% ± 1.00% *vs.* 35.67% ± 5.03%, *p* < 0.001, **Figure 2D**). However, in the nivolumab treatment group, the viability of B cells had no effect on NK cell function (K562 cells: 20.47% ± 5.25% *vs.* 17.80% ± 2.13%, *p* > 0.05, **Figure 2C**; Raji cells: 15.03% ± 0.95% *vs.* 11.30% ± 1.47%, *p* > 0.05, **Figure 2D**). The results indicate that living B cells are necessary during NK cell activation by non-coated rituximab.

We also found that non-coated rituximab induced much higher CD107a expression (K562 cells: 42.06% ± 12.35% *vs.* 30.44% ± 8.60%, *p* < 0.001; Raji cells: 24.43% ± 16.92% *vs.* 16.39% ± 11.14%, *p* < 0.001) in NK cells than in cells treated with coated rituximab (**Figures 2E–H**). These results indicate that NK cells obtained using non-coated rituximab had a greater cytotoxic effect on different tumor cells.

### NK Cells Activated by Non-Coated Rituximab Had Higher Levels of Perforin and Granzyme B Than Those Activated by Coated Rituximab

**Figure 3:**
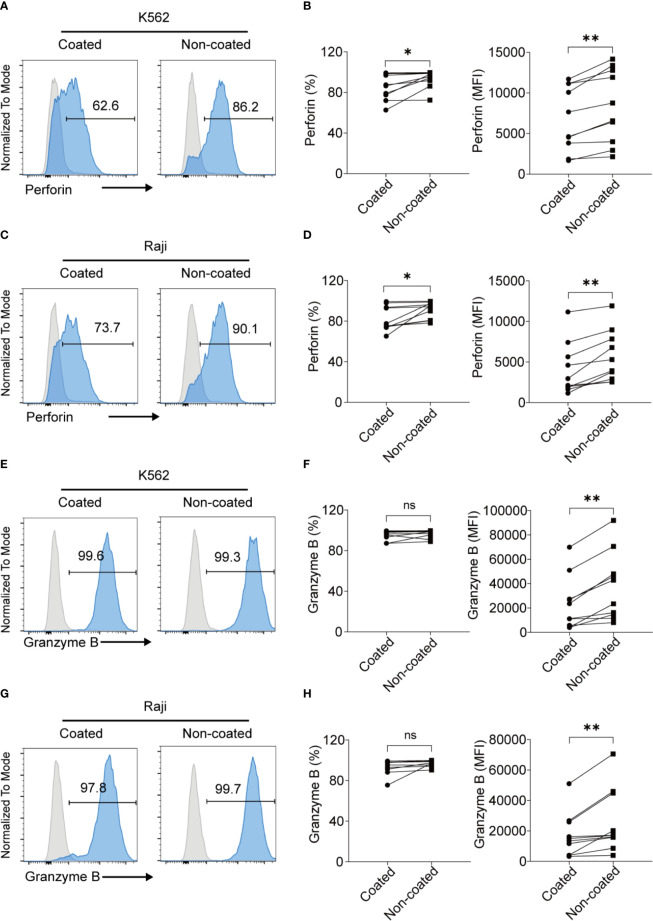
NK cells expanded with non-coated rituximab had higher levels of perforin and granzyme B expression. NK cells were collected after expansion for 14 d. **(A, C, E, G)** Flow cytometry analysis of perforin^+^ and granzyme B^+^ NK cells induced with coated or non-coated rituximab from one representative donor. NK cells were incubated with K562 or Raji cells for 4 h, followed by perforin and granzyme B staining. **(B, D, F, H)** Quantification of perforin^+^ and granzyme B^+^ NK cells activated with coated or non-coated rituximab. n = 10 donors, ns, not significant *p* > 0.05, *p < 0.05, **p < 0.01; two-tailed paired t-test.

NK cells can kill target cells *via* perforin and granzyme B. To further analyze the expression of perforin and granzyme B in NK cells obtained by the different methods, NK cells were co-cultured with NK cell-sensitive K562 cells and NK cell-resistant Raji cells for 4 h. The NK cells induced by non-coated rituximab were associated with higher perforin levels in K562 cells (percentage of positive cells: 93.44% ± 8.50% *vs.* 85.88% ± 12.84%, *p* < 0.05; mean fluorescence intensity [MFI]: 8308 ± 4542 *vs.* 6832 ± 3982, *p* < 0.01; **Figures 3A, B**) and Raji cells (percentage of positive cells: 91.11% ± 8.25% *vs.* 82.50% ± 12.14%, *p* < 0.05; MFI: 5647.00 ± 3159.00 *vs.* 4057 ± 3212, *p* < 0.01; **Figures 3C, D**) than NK cells cultured with coated rituximab. Although the percentages of granzyme B-positive NK cells induced by non-coated and coated rituximab were comparable (K562 cells: 96.74% ± 3.90% *vs.* 95.37% ± 4.73%, *p* > 0.05; Raji cells: 97.14% ± 3.08% *vs.* 93.63% ± 7.34%, *p* > 0.05), granzyme B MFI in the non-coated rituximab group was significantly higher than the control group (K562 cells: 37364 ± 27858 *vs.* 23512.00 ± 21873, *p* < 0.01; Raji cells: 26032 ± 20887 *vs.* 17159 ± 14525, *p* < 0.01; **Figures 3E–H**).

### NK Cells Activated by Non-Coated Rituximab Had Different Surface Markers Compared to Naïve NK Cells and NK Cells Activated by Coated Rituximab

**Figure 4:**
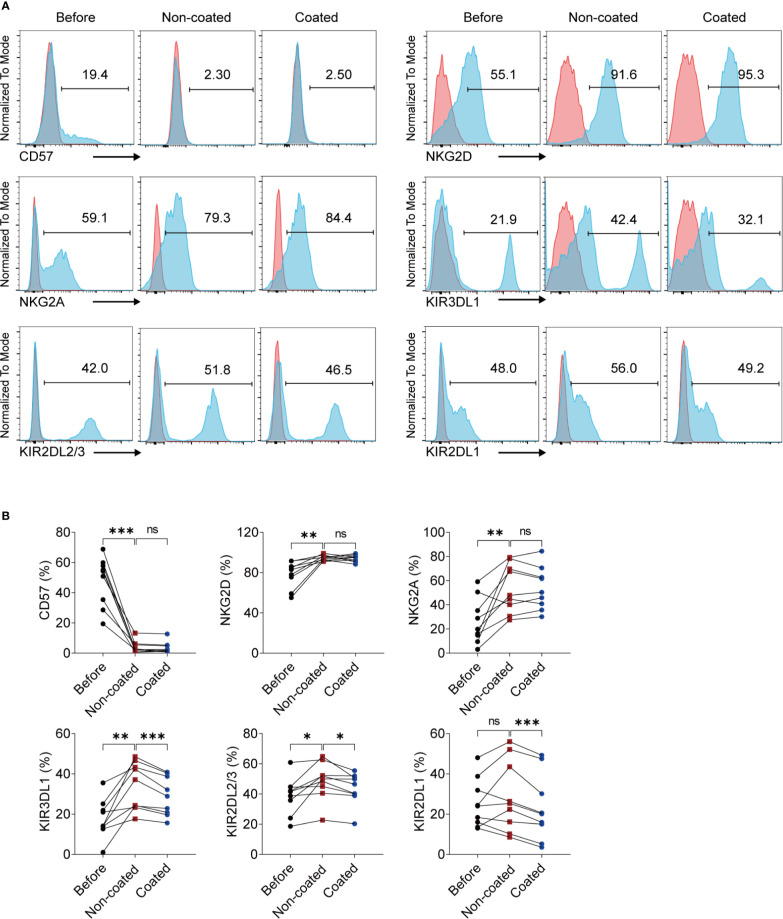
NK cells expanded by non-coated rituximab have different phenotypes compared to naïve NK cells and NK cells induced by coated rituximab. **(A)** Representative flow cytometry analysis of the percentages of CD57, NKG2D, NKG2A, KIR3DL1, KIR2DL2/3, and KIR2DL1 on the NK cells before and after induction by coated and non-coated rituximab. **(B)** Graphs showing the percentages of CD57, NKG2D, NKG2A, KIR3DL1, KIR2DL2/3, and KIR2DL1 on the NK cells before induction and after induction by coated and non-coated rituximab. n = 9 donors, ns, not significant, *p* > 0.05, *p < 0.05, **p < 0.01, ***p < 0.001; one-way ANOVA.

Some NK cell surface molecules are closely related to NK cell function, including CD57, NKG2D, NKG2A, and killer cell inhibitory receptors (KIRs). We measured changes of these molecules on NK cells before and after induction by non-coated rituximab. The results showed that the expression of CD57 (47.80% ± 16.28% vs. 4.06% ± 3.92%, *p* < 0.001) on the surface of expanded NK cells decreased significantly, while the expression of NKG2D (78.26% ± 13.17% *vs.* 94.57% ± 2.74%, *p* < 0.01), NKG2A (26.37% ± 18.88% *vs.* 53.94% ± 20.09%, *p* < 0.01), KIR3DL1 (17.69% ± 9.64% *vs.* 34.12% ± 11.80%, *p* < 0.01), and KIR2DL2/3 (38.98% ± 12.30% vs. 48.68% ± 12.43%, *p* < 0.05) significantly increased. However, there was no significant difference in the expression of KIR2DL1 (25.33% ± 12.08% *vs.* 28.97% ± 17.57%, *p* > 0.05) on NK cells before and after induction. We also compared the effects of coated and non-coated rituximab on the expression of these molecules on NK cells and found that expression of KIR2DL1 (28.97% ± 17.57% *vs.* 23.02% ± 16.43%, *p* < 0.001), KIR2DL2/3 (48.68% ± 12.43% *vs.* 43.77% ± 10.58%, *p* < 0.05), and KIR3DL1 (34.12% ± 11.80% *vs.* 28.89% ± 9.65%, *p* < 0.001) on NK cells induced by non-coated rituximab was significantly higher than in NK cells induced by coated rituximab (**Figure 4**).

### NK Cells Activated by Non-Coated Rituximab Had a Substantial IFN-γ Secretion Potential Compared to Those Activated by Coated Rituximab

**Figure 5:**
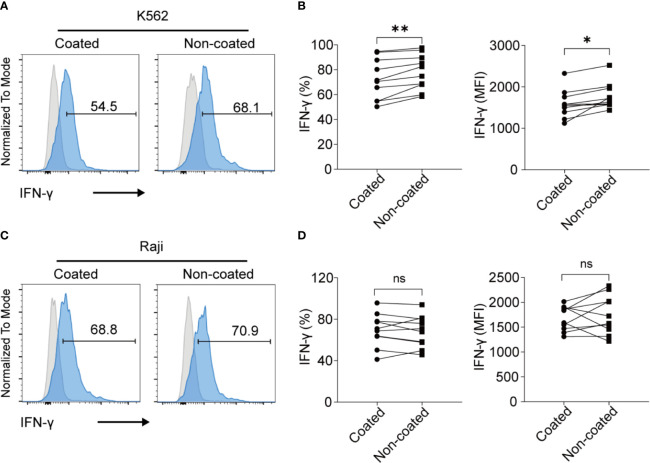
NK cells expanded with non-coated rituximab had a stronger IFN-γ secretion ability. After expansion for 14 d, the secretion of IFN-γ by NK cells was assayed. **(A, C)** Flow cytometry analysis of IFN-γ^+^ NK cells induced with coated or non-coated rituximab from one representative donor. NK cells were incubated with K562 cells or Raji cells for 4 h, followed by IFN-γ staining. **(B, D)** Quantification of IFN-γ^+^ NK cells activated with coated or non-coated rituximab. n = 10 donors, ns, not significant *p* > 0.05, *p < 0.05, **p < 0.01; two-tailed paired t-test.

When NK cells were incubated with K562 cells, the NK cells induced by non-coated rituximab had higher levels of IFN-γ (percentage of positive cells: 78.06% ± 14.21% vs. 72.41% ± 16.44%, *p* < 0.01; MFI: 1778 ± 316 vs. 1588 ± 342, *p* < 0.05; **Figures 5A, B**) than NK cells cultured with coated rituximab. However, after incubation with Raji cells, no significant difference was found in IFN-γ expression (percentage of positive cells: 68.19% ± 15.32% vs. 69.47% ± 16.05%, *p* > 0.05; MFI: 1746 ± 389.30 vs. 1683 ± 249.40, *p* > 0.05; **Figures 5C, D**) between NK cells obtained by either method.

### NK Cells Activated by Non-Coated Rituximab Had a Higher ADCC Effect

**Figure 6:**
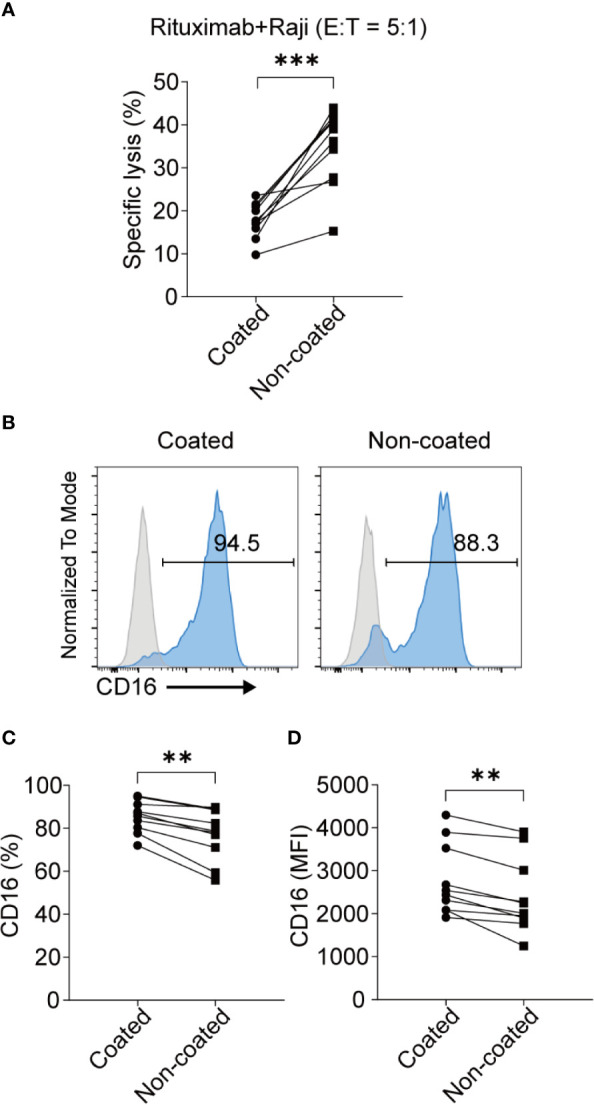
NK cells induced with non-coated rituximab had a stronger ADCC effect. NK cells were activated by coated and non-coated rituximab for 3 d. Culturing was then continued for an additional 11 d. The NK cells were collected and washed with PBS and the ADCC activity was determined by calcein-release assay. **(A)** NK cells incubated with calcein-AM-labeled Raji cells and rituximab for 4 h. NK cell cytotoxicity was determined by calcein-release assay. n = 10 donors, ***p < 0.001; two-tailed paired t-test. **(B)** Representative flow cytometry analysis of the percentage of CD16^+^ NK cells induced with coated or non-coated rituximab. **(C, D)** Statistical analyses of the percentages and MFI of CD16 in NK cells induced with coated or non-coated rituximab. n = 10 donors, **p < 0.01; two-tailed paired t-test.

ADCC is an important function of NK cells. We found that the NK cells obtained using non-coated rituximab had a significantly stronger ADCC effect (34.73% ± 9.00% *vs.* 17.73% ± 4.08%, *p* < 0.001; **Figure 6A**); however, the expression of CD16 on NK cells obtained with non-coated rituximab was lower compared to cells obtained with coated rituximab (percentage: 76.99% ± 11.86% *vs.* 85.48% ± 7.34%, *p* < 0.01; MFI: 2409.00 ± 871.60 vs. 2778.00 ± 831.00, *p* < 0.01; **Figures 6B–D**).

### Apoptosis and Cell-Cycle Progression in NK Cells Induced by Coated and Non-Coated Rituximab Did Not Differ

**Figure 7:**
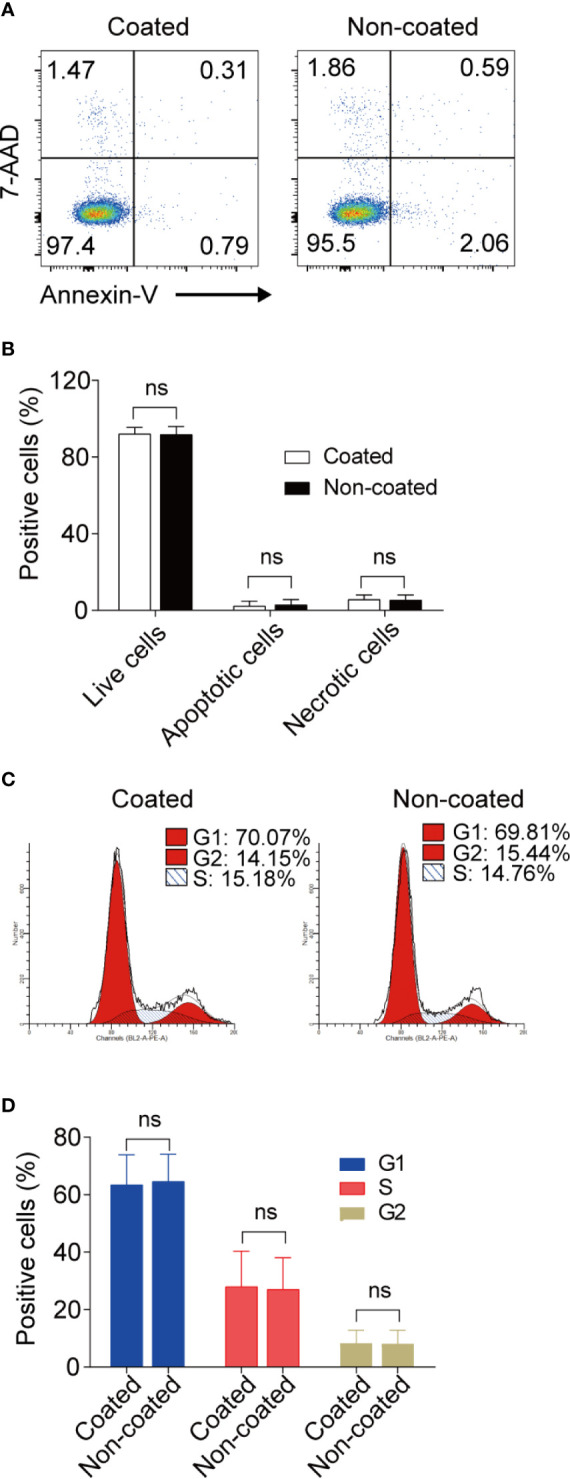
Apoptosis and cell-cycle progression in NK cells induced by coated and non-coated rituximab did not differ. After expansion for 14 d, the NK cells were collected and assayed for apoptotic activity and cell cycle status. **(A, C)** Representative flow cytometry analysis of the percentage of apoptotic cells and the cell-cycle progression of cultured NK cells with coated or non-coated rituximab. **(B, D)** Statistical analyses of the percentages of apoptotic cells and the cell-cycle progression of NK cells cultured with coated or non-coated rituximab. n = 10 donors, ns, not significant *p* > 0.05; two-tailed paired t-test.

No differences were found in terms of apoptosis and cell-cycle progression for the expanded NK cells between the non-coated rituximab group and coated rituximab group (**Figure 7**).

### Non-Coated Rituximab Activated NK Cells Through the AKT–XBP1s Axis

**Figure 8:**
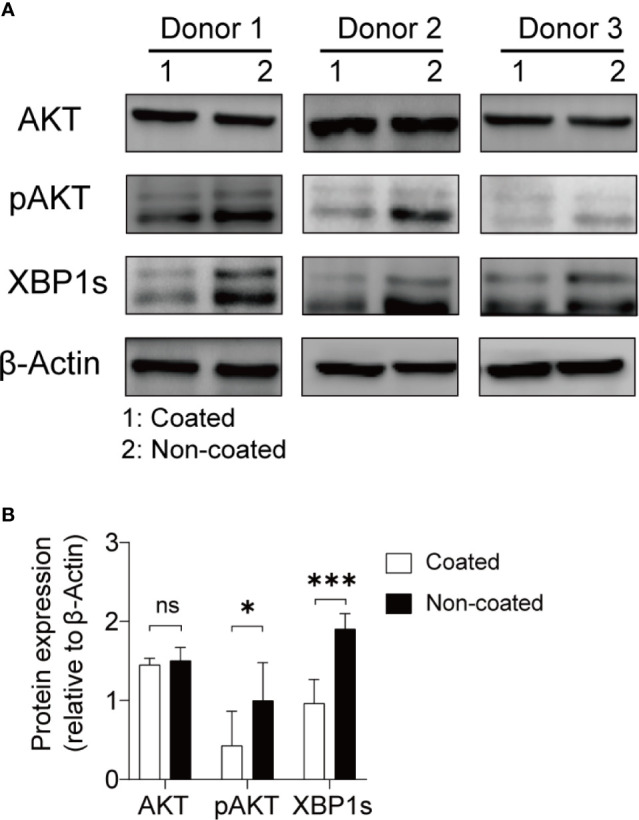
NK cells expanded with non-coated rituximab had higher levels of p-AKT and XBP-1s. After expansion for 14 d, NK cell proteins were extracted and assayed by western blotting. **(A)** Western blots for AKT, p-AKT, and XBP1s in NK cells from three donors activated with coated or non-coated rituximab. **(B)** Densitometric quantification showing the ratios of AKT, p-AKT, and XBP1s to β-actin. n = 3 donors, ns, not significant *p* > 0.05, *p < 0.05, ***p < 0.001; two-tailed paired t-test.

The AKT–XBP1s axis is an important regulatory pathway for NK cell function and is closely related to the release of cytokines, perforin, and granzyme by NK cells (29). We compared changes in the AKT–XBP1s axis in NK cells induced by coated and non-coated rituximab. Our results showed that AKT phosphorylation and XBP1s expression in NK cells from non-coated rituximab were significantly higher than those obtained in coated rituximab (**Figure 8**). These results further indicate that non-coated rituximab was more effective in activating NK cells.

## Discussion

NK cells are important innate immune cells which are closely related to tumorigenesis and metastasis (2). *In vitro* and *in vivo* studies have demonstrated that NK cells can exert a powerful antitumor function without pro-stimulation. NK cells can directly kill tumor cells by secreting perforin, granzyme B, and cytokines (30). The use of NK cell therapy is challenging due to the need to boost NK cell cytotoxicity. In this study, we explored a new method for inducing highly cytotoxic NK cells *via* autologous living B cells and rituximab. This method enhances the activity of NK cells and has the potential to improve the safety of NK cell therapy in clinical settings, both of which are crucial for clinical efficacy.

There are many ways to enhance the activity of NK cells (24). Using feeder cells is a very effective method for expanding and activating NK cells; however, as feeder cells are usually cancer cells, this approach increases the risks of NK cell therapy in clinical applications. Previously, we reported a method for activating NK cells using an anti-CD16 mAb without feeder cells, where coating the CD16 mAb on the cell culture flask efficiently activated NK cells. We found that coated rituximab can also be used to culture NK cells. In terms of NK cell cytotoxicity, NK cells activated with coated rituximab were similar to those coated with the CD16 mAb. However, the molecular mechanisms involved in NK activation by coated CD16 mAb and coated rituximab are clearly distinct. The CD16 mAb that we used is a mouse IgG1 with low binding capacity for human CD16, while rituximab is a human IgG1 with high affinity for CD16. The NK-activating activity of coated CD16 mAb will mostly be related to crosslinking of human CD16 *via* Fab binding. On the other hand, coated rituximab can activate NK cells only through an accessible Fc portion binding to CD16.

During *in vitro* NK cell expansion from PBMCs, B cells die spontaneously over time in the absence of CD40 stimulation or IL-4 (31). Rituximab, a mAb that targets CD20, can reduce the number of normal B cells through ADCC in clinical treatment, as normal B cells also express CD20 (32). In this study, we were interested in determining whether B cells can be used as feeder cells to activate NK cells through rituximab-mediated ADCC. To address this question, we depleted B cells from the NK cell-induction system and found that the antitumor function of NK cells activated by non-coated rituximab decreased sharply. However, the cytotoxicity of NK cells induced by coated rituximab was not affected by B cell elimination. After B cells were fixed, although the antitumor effect of NK cells activated by rituximab group was stronger than that of nivolumab group, it was still significantly lower than that of rituximab combined with living B cells group. Furthermore, rituximab F(ab’)2 fragments with living B cells did not elicit enhanced NK cytotoxicity (**Figure S1**), which revealed that cytokine secretion by rituximab-triggered B cells apparently plays no independent role. These results indicate that rituximab combined with living B cells can better activate NK cells through ADCC. CD16 is associated with the immunoreceptor tyrosine-based activation motif containing T-cell receptor ζ chain (33). NK cells activate upon the formation of the ζ-chain homodimer (34). We speculate that when the ADCC effect occurs among living B cells, rituximab and NK cells, synapses will be formed at the contact sites, and a large number of CD20 molecules will gather at the synapse sites to combine with rituximab and CD16 molecules, enhancing the formation of the ζ-chain homodimer in NK cells, thus leading to their activation. However, when B cells are fixed, the fluidity of the B cell membrane is weakened, which is not conducive to the formation of the ζ-chain homodimer in NK cells. Therefore, the activation of NK cells activated by fixed B cells and rituximab is obviously weakened.

**Figure 9:**
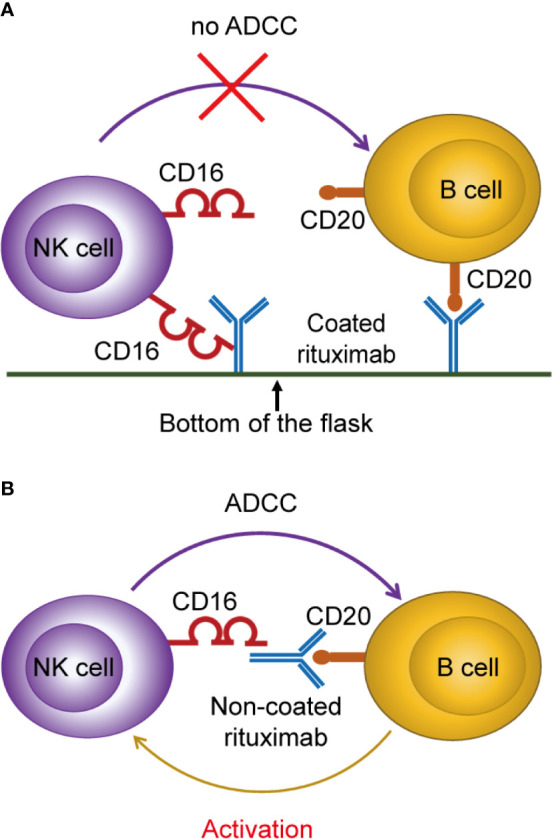
Proposed mechanism of NK cell activation and expansion by non-coated rituximab *via* B cells. **(A)** When rituximab is coated at the bottom of the flask, the Fc fragment of rituximab binds to the bottom surface of the flask, making it difficult to bind NK cells and B cells at the same time, and inhibiting the ADCC effect to activate NK cells. **(B)** When rituximab was not coated at the bottom of the flask, it could bind NK cells and B cells simultaneously and activate NK cells through the ADCC effect.

We found that NK cells cultured with non-coated rituximab showed stronger antitumor ability and higher expression levels of CD107a, granzyme B, and perforin comparing to NK cells cultured with coated rituximab. Therefore, we speculate that coated rituximab is not conducive to binding B cells and NK cells simultaneously because the Fc fragment is fixed at the bottom of the flask, which affects the exertion of the ADCC effect. Therefore, the effect of activating NK cells is weakened (**Figure 9A**). When rituximab is not coated at the bottom of the flask, non-coated rituximab can connect B cells to NK cells and exert the ADCC effect, thereby activating and expanding NK cells (**Figure 9B**).

CD57 is a marker of highly differentiated cells (35). The proliferative ability of CD57^+^ NK cells is lower than CD57^-^ NK cells (35, 36). We found that the expression of CD57 on NK cells activated by rituximab decreased significantly, which indicated that NK cells obtained by rituximab treatment still had strong proliferative ability. We also found that the expression of KIR2DL1, KIR2DL2/3, and KIR3DL1 on NK cells induced by non-coated rituximab was significantly higher than in NK cells induced by coated rituximab. It has been reported that mature anergic NK cells (expressing inhibitory KIR receptors for MHC-I) that are adoptively transferred into an MHC-I-sufficient setting become responsive (37). Therefore, we speculate that non-coated rituximab not only mediates NK cells to exert an ADCC effect on B cells, but also strengthens the interactions between KIR molecules on NK cells and MHC-I on B cells, making NK cells responsive. This may be the reason why the antitumor function of NK cells activated by non-coated rituximab was more robust.

CD16 expression on NK cells expanded using non-coated rituximab was lower than NK cells expanded with coated rituximab. Loss of CD16 has recently been shown to be important for NK cell detachment and sequential engagement of multiple target cells (38). Rapid detachment between NK cells and target cells increased engagement of NK cells and multiple targets and enabled a greater proportion of NK cells to perform serial killing. In addition, detachment of NK cells from target cells can avoid excessive activation of NK cells and ensure the survival of NK cells (39). Therefore, the shedding of CD16 may be an important molecule to keep NK cells containing high anti-tumor effect without over-activation. We also found that the ADCC effect and IFN-γ-secretion level in NK cells in the non-coated rituximab group were more robust. This finding further confirms that non-coated rituximab might be more suitable than coated rituximab to promote full NK cell activation. CD161, expressed on the surface of NK cells, combines with lectin-like transcript 1 expressed on the surface of B cells and transmits inhibitory signals to NK cells (40). To improve the rituximab-mediated activation of NK cells by B cells, an anti-CD161 mAb was added to the NK cell culture medium to block the transmission of inhibitory signals from B cells to NK cells. Rituximab and the CD161 mAb were only added during the first few days of culture because B cells are gradually consumed by NK cells as feeder cells during this time (**Figure S2**). Therefore, there was no need to add these antibodies during subsequent culturing. Although NK cells were activated by B cells and rituximab during the initial days of culture, they maintained high activity and did not undergo apoptosis after 14 d in culture. This finding indicates that the method developed in this study is effective in activating NK cells and promoting the amplification of activated NK cells. This method could be used to avoid excessive activation of NK cells by rituximab and reduce cell culture cost.

The engagement of CD16 molecules by mAbs has been reported to markedly induce AKT activation (41). In this study, we found that AKT phosphorylation in the non-coated rituximab group was higher than the coated rituximab group. Additionally, XBP1s accumulated at higher levels in NK cells induced with non-coated rituximab than in NK cells induced with coated rituximab. The AKT–XBP1s axis plays a critical role in the antitumor function of NK cells (29). Therefore, we speculate that non-coated rituximab efficiently combines with B cells and CD16 molecules, increasing AKT activation and the abundance of XBP1s, which then activate NK cells.

In conclusion, we established a new method for activating and amplifying NK cells with non-coated rituximab *via* autologous living B cells from PBMCs, that showed a potent antitumor function compared to NK cells induced with coated rituximab. This method not only simplifies technical operation, but also enhances the safety of NK cell therapy, which will benefit the use of this as a treatment in the clinic.

## Data Availability Statement

The original contributions presented in the study are included in the article/**Supplementary Material**. Further inquiries can be directed to the corresponding authors.

## Ethics Statement

The studies involving human participants were reviewed and approved by The ethics committee of the First Hospital of Jilin University. The patients/participants provided their written informed consent to participate in this study.

## Author Contributions

JC, WL, and YW conceived the study. CN and SZ designed and carried out part of the experiments and drafted the entire manuscript. YC, ML, LZ, DX, ZL, and JX carried out part of the experiments. All authors contributed to the article and approved the submitted version.

## Funding

This work was supported by grants from the National Key Research and Development Program of China (grant number 2020YFA0707704), the National Natural Science Foundation of China (grant numbers 31700764, 81972684, and 81702589), the Jilin Provincial Science and Technology Department (grant numbers 20200201180JC, 20200602032ZP, 20180101009JC, and 20190303146SF), the Scientific and Technological Research of Jilin Provincial Education Department (grant number JJKH20190023KJ), the Jilin Province Finance Department (grant number 2018SCZWSZX-010), the Science and Technology Achievement Transformation Fund Project of the First Hospital of Jilin University (grant numbers JDYYZH-1902037 and JDYYZH-1902038), and the China Guanghua Foundation & First Hospital of Jilin University (grant numbers JDYYGH2019004 and JDYYGH2019012).

## Conflict of Interest

The authors declare that the research was conducted in the absence of any commercial or financial relationships that could be construed as a potential conflict of interest.

## Supplementary Material

Supplementary Figure 1 F(ab’)2 fragments have no effect on the antitumor function of NK cells. Rituximab was treated with pepsin to obtain the F(ab’)2 fragments of rituximab. Then rituximab and the F(ab’)2 fragments were used for NK cell culture, and PBS was used as a negative control. After 14 d in culture, cells were harvested and cytotoxicity was determined by calcein-release assay. n = 3 donors, ns: not significant, p > 0.05; one-way ANOVA.

Supplementary Figure 2 Changes in the proportion of NK cells, B cells, CD3^+^CD8^+^ T cells, and CD3^+^CD4^+^ T cells during NK activation and expansion *in vitro*. **(A)** Representative flow cytometry analysis of the percentage of NK cells, CD19^+^ B cells, CD3^+^CD8^+^ T cells, and CD3^+^CD4^+^ T cells during the course (d0, d3, d7, d14) of NK cell culture with non-coated rituximab. **(B)** Statistical analyses of the percentages of NK cells, CD19^+^ B cells, CD3^+^CD8^+^ T cells, and CD3^+^CD4^+^ T cells during NK cell culture with non-coated rituximab. n = 9 donors, ns: not significant, *p* > 0.05, ****p* < 0.001; one-way ANOVA.

## Abbreviations

ADCC, antibody-dependent cell-mediated cytotoxicity; AKT, protein kinase B; KIR, killer cell inhibitory receptors; IFN, interferon; IL, interleukin; mAb, monoclonal antibody; MFI, mean fluorescence intensity; NK, natural killer; PBMC, peripheral blood mononuclear cell; XBP1s, X-box binding protein 1, short isoform.
