# Supplementary figures and images for: Non-Coated Rituximab Induces Highly Cytotoxic Natural Killer Cells From Peripheral Blood Mononuclear Cells via Autologous B Cells
Source: Front Immunol. 2021 May 25;12:658562. doi: 10.3389/fimmu.2021.658562 (PMC8185348; doi:10.3389/fimmu.2021.658562)

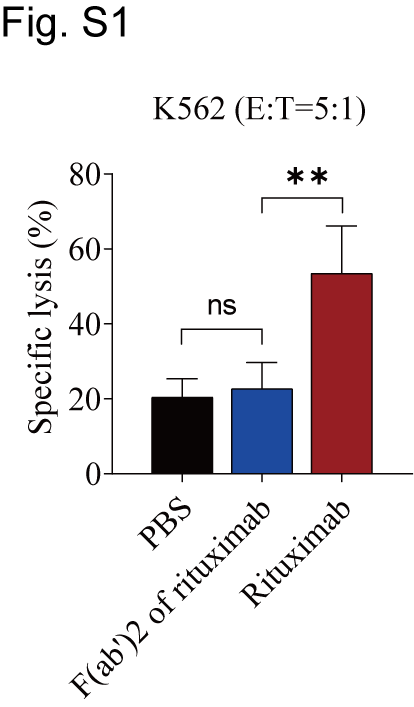

Supplement: Supplementary Figure 1 — F(ab’)2 fragments have no effect on the antitumor function of NK cells. Rituximab was treated with pepsin to obtain the F(ab’)2 fragments of rituximab. Then rituximab and the F(ab’)2 fragments were used for NK cell culture, and PBS was used as a negative control. After 14 d in culture, cells were harvested and cytotoxicity was determined by calcein-release assay. n = 3 donors, ns: not significant, p > 0.05; one-way ANOVA. [file Image_1.tif]

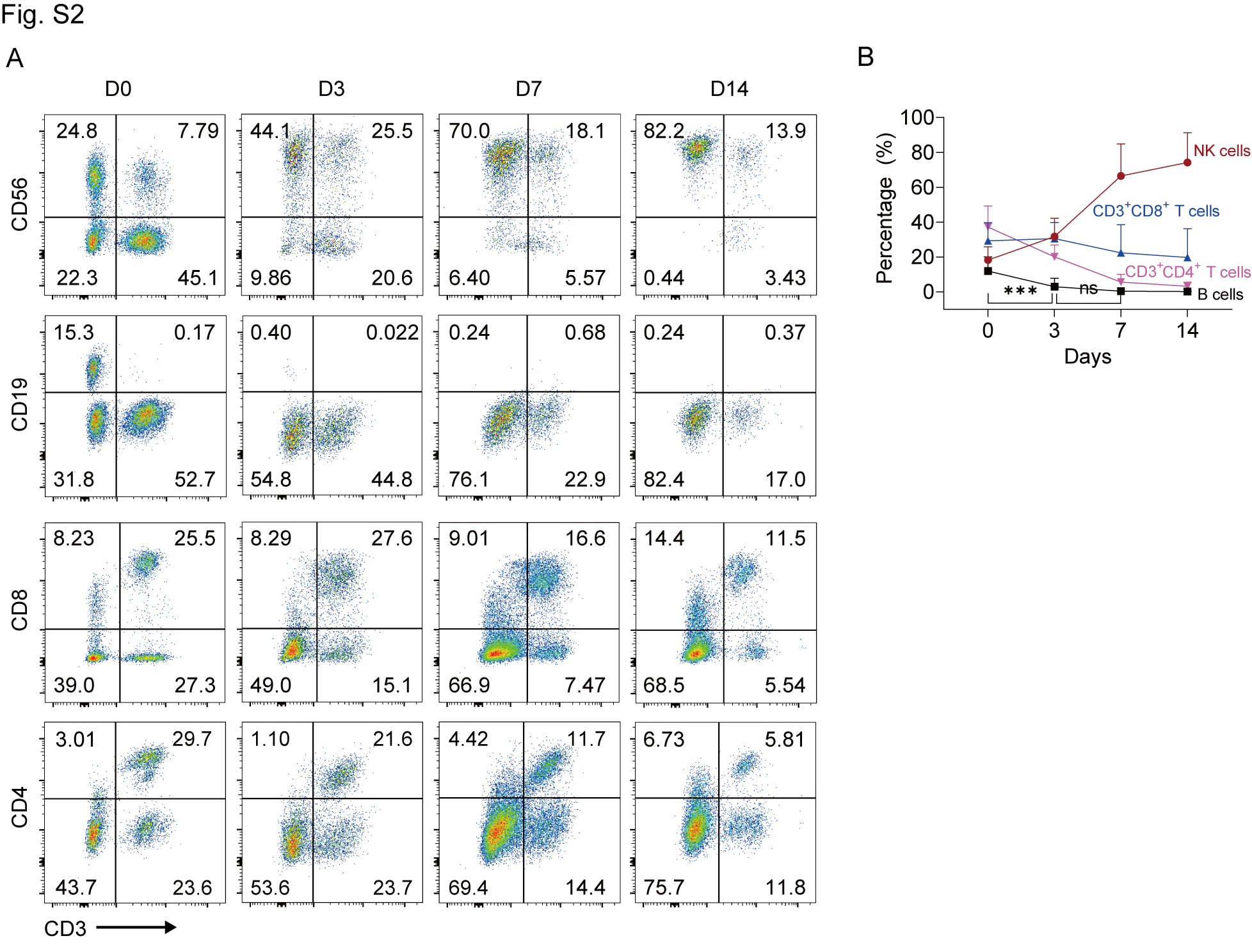

Supplement: Supplementary Figure 2 — Changes in the proportion of NK cells, B cells, CD3+CD8+ T cells, and CD3+CD4+ T cells during NK activation and expansion in vitro. (A) Representative flow cytometry analysis of the percentage of NK cells, CD19+ B cells, CD3+CD8+ T cells, and CD3+CD4+ T cells during the course (d0, d3, d7, d14) of NK cell culture with non-coated rituximab. (B) Statistical analyses of the percentages of NK cells, CD19+ B cells, CD3+CD8+ T cells, and CD3+CD4+ T cells during NK cell culture with non-coated rituximab. n = 9 donors, ns: not significant, p > 0.05, ***p < 0.001; one-way ANOVA. [file Image_2.tif]
